# Two‐year trends from the LANDMARC study: A 3‐year, pan‐India, prospective, longitudinal study on the management and real‐world outcome in patients with type 2 diabetes mellitus

**DOI:** 10.1002/edm2.404

**Published:** 2023-02-01

**Authors:** Ashok K. Das, Sanjay Kalra, Shashank Joshi, Ambrish Mithal, Prasanna Kumar K. M., Ambika G. Unnikrishnan, Hemant Thacker, Bipin Sethi, Subhankar Chowdhury, Amarnath Sugumaran, Senthilnathan Mohanasundaram, Shalini K. Menon, Vaibhav Salvi, Deepa Chodankar, Saket Thaker, Chirag Trivedi, Subhash K. Wangnoo, Abdul H. Zargar, Nadeem Rais

**Affiliations:** ^1^ Pondicherry Institute of Medical Sciences Puducherry India; ^2^ Bharti Hospital Karnal India; ^3^ Lilavati Hospital Mumbai India; ^4^ Medanta‐ The Medicity Gurgaon India; ^5^ Centre for Diabetes and Endocrine Care Bengaluru India; ^6^ Chellaram Diabetes Institute Pune India; ^7^ Bhatia Hospital Mumbai India; ^8^ Care Hospital Hyderabad India; ^9^ IPGME and R and SSKM Hospital Kolkata India; ^10^ Sanofi Mumbai India; ^11^ Apollo Hospital Education and Research Foundation New Delhi India; ^12^ Center for Diabetes and Endocrine Care Srinagar India; ^13^ Chowpatti Medical Centre Mumbai India

**Keywords:** cardiovascular diseases, diabetes complications, diabetic nephropathies, glycaemic control

## Abstract

**Introduction:**

There are limited data on the real‐world management of diabetes in the Indian population. In this 2‐year analysis of the LANDMARC study, the management of type 2 diabetes mellitus (T2DM) and related complications were assessed.

**Method:**

This multicenter, observational, prospective study included adults aged ≥25 to ≤60 years diagnosed with T2DM (duration ≥2 years at enrollment) and controlled/uncontrolled on ≥2 anti‐diabetic agents. This interim analysis at 2 years reports the status of glycaemic control, diabetic complications, cardiovascular (CV) risks and therapy, pan‐India including metropolitan and non‐metropolitan cities.

**Results:**

Of the 6234 evaluable patients, 5318 patients completed 2 years in the study. Microvascular complications were observed in 17.6% of patients (1096/6234); macrovascular complications were observed in 3.1% of patients (195/6234). Higher number of microvascular complications were noted in patients from non‐metropolitan than in metropolitan cities (*p* < .0001). In 2 years, an improvement of 0.6% from baseline (8.1%) in mean glycated haemoglobin (HbA1c) was noted; 20.8% of patients met optimum glycaemic control (HbA1c < 7%). Hypertension (2679/3438, 77.9%) and dyslipidaemia (1776/3438, 51.7%) were the predominant CV risk factors in 2 years. The number of patients taking oral anti‐diabetic drugs in combination with insulin increased in 2 years (baseline: 1498/6234 [24.0%] vs. 2 years: 1917/5763 [33.3%]). While biguanides and sulfonylureas were the most commonly prescribed, there was an evident increase in the use of dipeptidyl peptidase‐IV inhibitors (baseline: 3049/6234, 48.9% vs. 2 years: 3526/5763, 61.2%).

**Conclusion:**

This longitudinal study represents the control of T2DM, its management and development of complications in Indian population.

**Clinical Trial Registration Number:**

CTRI/2017/05/008452.

## Graphical abstract

This 2‐year interim analysis of the 3‐year long LANDMARC study highlights the T2DM burden, management practices and related complications, across metropolitan and non‐metropolitan cities of India. It indicates that the burden of uncontrolled T2DM in India is high with 20.8% of participants achieving glycaemic control in 2 years (HbA1c < 7%; 53 mmol/mol); and 17.6% having microvascular and 3.1% having macrovascular complications. There is need for effective diabetes management to meet glycaemic targets and prevent CV risk and vascular complications in a developing country like India with high prevalence of T2DM.

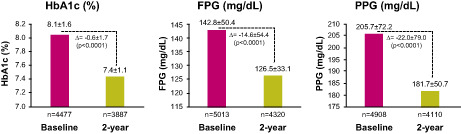

## 1 INTRODUCTION

India has been severely affected by the global diabetes epidemic. As per the 10th edition of International Diabetes Federation's (IDF) diabetes atlas (2021), India has 74.2 million people living with diabetes currently, with an age‐adjusted prevalence of 9.6% among adults. India is expected to have 124.9 million people in the age range of 20–79 years living with diabetes by 2045.^1^

The American Diabetes Association (ADA) recommends a combination of modified lifestyle and pharmacological treatment to achieve good metabolic control in diabetes and long‐term maintenance.^2^, ^3^ Earlier studies have demonstrated that baseline glycated haemoglobin (HbA1c) and body mass index (BMI) can serve as important biomarkers to understand the disease aetiology and to identify suitable treatment options.^4^, ^5^

Long‐term uncontrolled diabetes can cause cardiovascular (CV) diseases and damage kidneys, nerves and other vital organs. If optimum diabetes control is achieved, these serious complications can be delayed or prevented altogether.^1^ This has also been substantiated by a study demonstrating that the occurrence of variations in glycaemic levels was associated with microvascular and macrovascular complications.^6^ The INSPIRED study, which was conducted in India, included 19,084 individuals (aged 10–97 years) with type 2 diabetes mellitus (T2DM) and varying phenotypic characteristics. The findings of this study emphasized the association between increased hazards of retinopathy and nephropathy with a rise in blood glucose levels.^7^ The Million death study also conducted in India showed that diabetes was associated with a significantly increased odds of stroke mortality (odds ratio, 95% confidence intervals [CI]: 1.6, 1.4–1.7, *p* < .0001).^8^ The American College of Cardiology (ACC)/American Heart Association (AHA)/Heart Failure Society of America (HFSA) guideline for the management of heart failure states that diabetes and heart failure often occur concomitantly, and each disease independently increases the risk of the other.^9^ Additionally, a review of clinical evidence‐based research on the association between T2DM and myocardial infarction (MI) showed that not only T2DM is strongly associated with MI, but it also increases the risk of developing MI and related complications.^10^ The review further discusses that in people with T2DM, MI is the primary cause of death and that T2DM leads to an increase in the risk of coronary events in individuals both with or without previous history of coronary events.^10^ Hence, tight glycaemic control is essential in the early stages of diabetes.

The LANDMARC study (LongitudinAl Nationwide stuDy on Management And Real‐world outComes of diabetes in India) was a 3‐year comprehensive, robust, longitudinal and prospective study. It aimed to collect data on glycaemic therapy and diabetes complications in people with diabetes living in different regions of India (including metropolitan and non‐metropolitan cities). The study protocol,^11^ baseline data^12^ and 1‐year results^13^ have been published earlier. The 1‐year results of the LANDMARC study indicate the progression of vascular complications and accumulation of CV risk among Indian patients with T2DM. Hypertension and dyslipidaemia, the most common CV risk factors reported, were pronounced in those who were overweight/had obesity. About one‐fifth of the patients had optimal glycaemic control (HbA1c < 7%). Patients from both non‐metropolitan and metropolitan cities were comparable in terms of improvement in glycaemic status and having optimum control.^13^

The aim of this 2‐year interim analysis was to further evaluate and understand the diabetic complications and T2DM management pattern in adult patients with T2DM across India (including a sub‐analysis of metropolitan and non‐metropolitan cities).

## 2 MATERIALS AND METHODS

### 2.1 Study design

This was a multicenter, observational, prospective study conducted over 3 years (conducted between March 2017 and July 2021). The study was divided into seven visits with an interval of 6 months each. The present manuscript includes results from the second year (within a window period of ±90 days) of the 3‐year evaluation period.

### 2.2 Study patients

Adults aged between ≥25 years and ≤60 years with T2DM for ≥2 years and who were controlled/uncontrolled on ≥2 anti‐diabetic agents at the time of enrollment were included in the study. The details of the study design, methodology, inclusion/exclusion criteria and statistical analysis have been published previously.^11^, ^12^, ^13^

### 2.3 Study assessments

At the end of the second year (visit 5), data related to glycaemic control status (fasting plasma glucose [FPG], post‐prandial glucose [PPG] and HbA1c) were collected. The proportion of patients with macrovascular complications (non‐fatal MI, non‐fatal stroke, CV death and peripheral vascular disease [PVD]), microvascular complications (retinopathy, nephropathy and neuropathy) and CV risk factors (hypertension, dyslipidaemia and albuminuria) was assessed. The glycaemic parameters and complications in patients from metropolitan and non‐metropolitan cities were assessed. The proportion of patients taking oral anti‐diabetic drugs (OADs) and injectable glucose‐lowering drugs was also assessed. Data related to anthropometry (weight) and frequency and severity of hypoglycaemia episodes were collected.

### 2.4 Data collection

The data related to the study end‐points were collected prospectively every 6 months up to the end of the study at 36 months. The 450 sites that were selected for this study represent the four geographical regions (East, West, North and South), urban/rural practice across India. The study design was planned to mirror real‐life management of patients with T2DM; hence, none of the assessments were mandated. The available data were recorded in electronic‐Case Report Forms (e‐CRFs). Data quality control was performed by qualified designated personnel. An adverse drug reaction related to any Sanofi product (clinical signs, laboratory values or other) was reported and followed up until the clinical recovery was complete and laboratory results (if clinically significant) had returned to normal or until progression had been stabilized. This was a planned interim analysis to assess the changes in the disease characteristics from baseline and may require modification in the assessment parameters for subsequent interim and final analyses.

### 2.5 Statistics

A minimum sample size of 4387 was decided assuming that the percentage of patients with composite incidence of non‐fatal MI, stroke and CV death after 3 years would have been 3%. The study planned to evaluate 6300 patients to estimate the composite incidence percentage with a precision of at least 1%, considering a 30% rate of patients dropping out from the study before the end of the 3 years.

### 2.6 Ethics

The protocol complies with the Declaration of Helsinki and this study was conducted in accordance with the principles laid by the 18th World Medical Assembly (Helsinki, 1964) and all subsequent amendments. The study was also in accordance with the guidelines for Good Epidemiology Practice (US & European)^14^, ^15^ and aligned to the local regulations, ethics committee(s) (institutional review board/independent ethics committee) and competent authorities. The study was approved by the ethics committees of all participating sites (or a central ethics committee, where applicable). All the patients provided written informed consent before data collection/documentation.

## 3 RESULTS

### 3.1 Demographics and baseline characteristics

**FIGURE 1:**
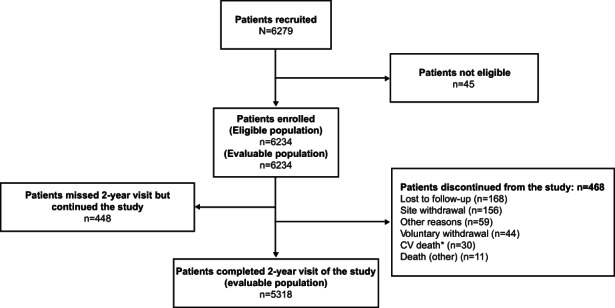
Patient disposition. *Reasons for CV death were sudden death (*n* = 19), myocardial infarction (*n* = 9), stroke (*n* = 1) and coronary artery procedure (*n* = 1). CV, cardiovascular; *n*, number of patients.

Among the 450 sites, data from 382 sites were analysed for the 2‐year visit. Of the 6279 patients recruited, 6234 patients were evaluated; of these, 5318 patients completed 2 years in the study (Figure 1). At baseline, the mean ± standard deviation (SD) age of the patients was 52.1 ± 9.2 years with 57.0% (3552/6234) of the study population in the age range of 50 to 65 years; more than half of the patients (56.6%, 3526/6234) were men. The mean ± SD baseline BMI was 27.2 ± 4.6 kg/m^2^, and the majority of the patients were obese (66.8%, 4149/6215) (Table 1). Most of the patients (74.4%, 4640/6234) were taking only OADs. (Table S1).

**TABLE 1:** Demographics and baseline characteristics.

Parameters	Baseline values
Age (years), *n*	6234
Mean ± SD (years)	52.1 ± 9.2
≤30 years	61 (1.0)
31–49 years	2192 (35.2)
50–65 years	3552 (57.0)
≥66 years	429 (6.9)
Gender, *n*	6234
Men	3526 (56.6)
Women	2708 (43.4)
Body mass index, *n*	6215
Mean ± SD (kg/m^2^)	27.2 ± 4.6
Underweight (<18.0)	44 (0.7)
Normal (18.0–22.9)	903 (14.5)
Overweight (23.0–24.9)	1119 (18.0)
Obese (≥25.0)	4149 (66.8)
Treatment at baseline, *n*	6234
Insulin	1549 (24.8)
Insulin‐naïve	4685 (75.2)
HbA1c (%), *n*	4477
Mean ± SD (%)	8.1 ± 1.6
<6.5%	528 (11.8)
6.5%–6.9%	593 (13.2)
<7%	1121 (25.0)
7%–7.9%	1420 (31.7)
8%–8.9%	922 (20.6)
≥9%	1014 (22.6)
Fasting plasma glucose, *n*	5013
Mean ± SD (mg/dl)	142.8 ± 50.4
Postprandial glucose, *n*	4908
Mean ± SD (mg/dl)	205.7 ± 72.3
Duration of T2DM (years)	
Mean ± SD	8.6 ± 5.6
Median (range)	7.1 (2.0, 40.7)
Duration of T2DM by treatment (years), mean ± SD	
Insulin, (*n* = 1549)	11.3 ± 6.6
Insulin‐naïve, (*n* = 4685)	7.7 ± 5.0

*Note*: Values are presented as *n* (%) unless specified otherwise. HbA1c was not measured for all patients and, hence, the percentage may not add up to 100%.

Abbreviations: HbA1c, glycated haemoglobin; *N*, number of patients analysed; *n*, number of patients with non‐missing results at the visit; SD, standard deviation; T2DM, type 2 diabetes mellitus.

### 3.2 Glycaemic status

**FIGURE 2:**
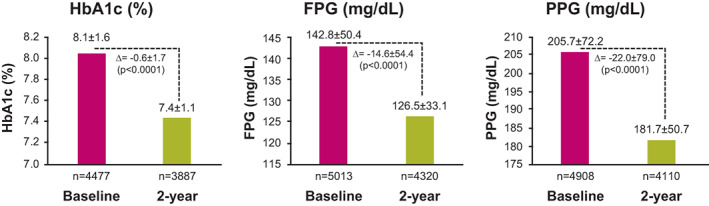
Change in glycaemic parameters at the end of 2 years. Values are presented as mean ± standard deviation. For change from baseline, HbA1c: *n* = 3020, FPG: *n* = 3668, PPG: *n* = 3454. FPG, fasting plasma glucose; HbA1c, glycated haemoglobin; *n*, number of patients analysed; PPG, postprandial glucose.

**FIGURE 3:**
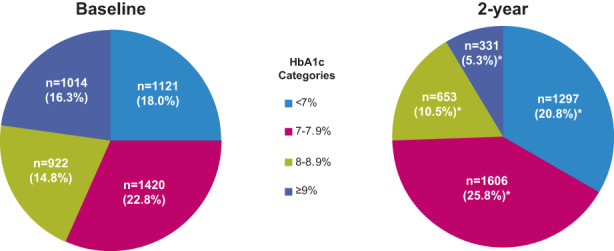
Proportion of patients across HbA1c categories (*N* = 6234). Data presented as *n* (%) from baseline (*N* = 6234). HbA1c was not measured for all patients and hence the percentage may not add up to 100%.*p*‐values are reported using McNemar's test with the null hypothesis that the proportion of paired samples is equal. Patients who met each criterion and those who did not meet the criteria are considered as binary outcomes for the test. The p‐values reported are not adjusted for inflation in type I error. *p < .0001. HbA1c, glycated haemoglobin; *N*, number of patients analysed; *n*, number of patients with non‐missing results at the visit.

In 2 years, all the glycaemic parameters improved (decreased) significantly from baseline (mean change ± SD: HbA1c: −0.6 ± 1.7%; FPG: −14.6 ± 54.5 mg/dl; and PPG: −22.0 ± 79.0 mg/dl; *p* < .0001) (Figure 2). When patients were stratified by HbA1c levels, a significant (*p* < .0001) reduction in the number of patients in the HbA1c 8%–8.9% and HbA1c ≥ 9% subgroups were noted at the 2‐year visit compared with baseline; while a significant (*p* < .0001) increase in patient numbers in the HbA1c 7%–7.9% subgroup was noted. Overall, 20.8% (1297/6234) of the patients met optimum glycaemic control (HbA1c < 7%) in 2 years (Figure 3).

### 3.3 Microvascular and macrovascular complications in 2 years

Microvascular complications were noted in 17.6% of patients (1096/6234) (Table 2); while new macrovascular complications were noted in 3.1% of patients (195/6234) (Table 3). The most frequently noted microvascular complication was neuropathy, which was reported in 0.6% of patients (32/6234), followed by nephropathy in 0.3% of patients (19/6234) and retinopathy in 0.1% of patients (6/6234) (Table 2). Overall, 57 new events of microvascular complications were reported in 55 patients at the 2‐year visit. Nephropathy, neuropathy and retinopathy were significantly (*p* < .0001) higher in patients with CV risk factors, while retinopathy was found to be significantly (*p* = .0351) higher in the HbA1c ≥ 7% subgroup. (Table S2).

**TABLE 2:** Proportion of patients with microvascular complications at baseline and 2 years (*N* = 6234).

Microvascular complications	Baseline *n* (%)	2 years *n* (%)	Patients at risk with new complications until 2 years, *n*	Patients with new complications at 2 years *n* (%)
Total microvascular complications	902 (14.5)	1096 (17.6)	6234	55 (0.9)
Neuropathy	737 (81.7)	898 (81.9)	5368	32 (0.6)
Nephropathy	154 (17.1)	221 (20.2)	6032	19 (0.3)
Retinopathy	141 (15.6)	165 (15.1)	6075	6 (0.1)

*Note*: This is an interim analysis and possible modifications on variables, and data could be performed for the subsequent interim analyses and final analysis. Abbreviations: *N*, number of patients analysed; *n*, number of patients with non‐missing results at the visit.

**TABLE 3:** Proportion of patients with macrovascular complications at baseline and 2 years (*N* = 6234).

Macrovascular complications	Baseline *n* (%)	2 years *n* (%)	Patients at risk with new complications until 2 years, *n*	Patients with new complications at 2 years *n* (%)
Total macrovascular complications	145 (2.3)	195 (3.1)	6234	15 (0.2)
Myocardial infarction	74 (51.0)	80 (41.0)	6234	1 (0.0)
Peripheral vascular disease	45 (31.0)	61 (31.3)	6234	4 (0.1)
Stroke	30 (20.7)	33 (16.9)	6234	1 (0.0)
Cardiovascular death	0	30 (15.4)	6213	9 (0.1)

*Note*: Values are presented as *n* (%) unless specified otherwise. This is an interim analysis and possible modifications on variables, and data could be performed for the subsequent interim analyses and final analysis. At 2 years, the newly documented macrovascular complications are reported. Abbreviations: *N*, number of patients analysed; *n*, number of patients with non‐missing results at the visit.

In 2 years, the most reported new macrovascular complications included CV death (0.1%, 9/6234) and PVD (0.1%, 4/6234) (Table 3). A total of 41 deaths were reported; of which, 30 deaths were attributed to CV causes (sudden death [*n* = 19], MI [*n* = 9], stroke [*n* = 1] and coronary artery procedure [*n* = 1]) and remaining 11 deaths were due to other causes (Figure 1). Three patients were hospitalized between the 1‐year to 18‐month period due to MI (one patient) and acute coronary syndrome (ACS; two patients). One patient was hospitalized due to ACS, heart failure and unstable angina between the 18‐month to 2‐year period (Table S3).

### 3.4 Cardiovascular risk factors

An increasing trend in the CV risk profile of patients was observed (baseline: 52.6%, 3281/6234 vs. 2‐year: 55.1%, 3438/6234). Among the 63 new cases (69 events) of CV risk factors, dyslipidaemia and hypertension (33 cases, each) were the most commonly reported (Table 4). Hypertension was noted more in men than women (*p* = .0019). Patients with hypertension and dyslipidaemia were greater in the subgroup having BMI ≥ 23 kg/m^2^ versus BMI < 23 kg/m^2^ (*p* < .0001 and *p* = .0525, respectively). Patients having hypertension and dyslipidaemia were higher in the subgroup having uncontrolled HbA1c levels (≥7%) versus controlled HbA1c levels (<7%) (Table 4).

**TABLE 4:** Summary of cardiovascular risk factors at 2‐year visit, by HbA1c, BMI and gender (*N* = 6234).

CV risk factors	Total *N* = 6234			
	Baseline	2 years	Patients at risk with new CV risk factors until 2 years, *n*	Patients with new CV risk factors at 2 years
Total number of CV risk factors, Ne	4419	4698		69
Patients with CV risk factors	3281 (52.6)	3438 (55.1)	6234	63 (1.0)
Hypertension^b^	2566 (78.2)	2679 (77.9)	3588	33 (0.9)
Dyslipidaemia^b^	1635 (49.8)	1776 (51.7)	4491	33 (0.7)
Albuminuria^b^	153 (4.7)	169 (4.9)	6234	3 (0.0)
Family history of PCD^b^	65 (2.0)	65 (1.9)	‐	‐
No complications	2562	‐	‐	‐
Unknown^c^	391	‐	‐	‐

CV risk factors by HbA1c, BMI and gender									
Risk factors	HbA1c < 7%	HbA1c ≥ 7%	*p*‐value^a^	BMI < 23 kg/m^2^	BMI ≥ 23 kg/m^2^	*p*‐value^a^	Men	Women	*p*‐value^a^
Hypertension	539 (8.7)	1135 (18.2)	.1787	264 (4.2)	1808 (29.0)	<.0001	1455 (23.3)	1224 (19.6)	.0019
Dyslipidaemia	352 (5.7)	807 (13.0)	.0098	191 (3.1)	1192 (19.1)	.0525	991 (15.9)	785 (12.6)	.4441
Albuminuria	2 (0.0)	0 (0.0)	.1113	0 (0.0)	3 (0.1)	>.9999	0	3 (0.1)	.0819
F/H of PCD	9 (0.1)	29 (0.5)	.2033	5 (0.1)	44 (0.7)	.3112	40 (0.6)	25 (0.4)	.4157

*Note*: Values are presented as *n* (%) unless specified otherwise.

Abbreviations: BMI, body mass index; CV, cardiovascular; F/H, family history; HbA1c, glycated haemoglobin; *N*, number of patients analysed; *n*, number of patients with non‐missing results at the visit; Ne, number of events; PCD, premature coronary disease.

^a^ *p*‐values are reported from Fisher's test if the cell frequency is lesser than 5. *p*‐values are reported using the Chi‐square test otherwise. The null hypothesis is that there is no difference between the two population proportions. The *p*‐values reported are not adjusted for inflation in type I error.

^b^ Percentages are calculated at baseline based on *N* = 3281 and at 2‐year visit based on *N* = 3438.

^c^ Patients who had chosen “No” and “Unknown” for multiple complications are counted under “Unknown”.

### 3.5 Glycaemic trends and vascular complications in metropolitan and non‐metropolitan cities

The baseline age, disease duration and HbA1c parameters were comparable across patients of non‐metropolitan and metropolitan cities (Table S4A). In 2 years, an improvement (decrease) was noted in all the glycaemic parameters (HbA1c, FPG and PPG) in patients from both metropolitan and non‐metropolitan cities (Table S4A).

The microvascular complications (neuropathy, nephropathy and retinopathy) were significantly (*p* < .0001) higher in patients from non‐metropolitan than in metropolitan cities (Table S4B). The number of CV deaths was higher in patients from non‐metropolitan than in metropolitan cities (19.7%, 25/135 vs. 7.4%, 5/70). Of the newly reported cases of macrovascular complications in the second year, in non‐metropolitan cities, PVD was reported in four patients, MI in one patient, CV death in eight patients and stroke in one patient; while in metropolitan cities, one new case was reported (CV death) (Table S4C). Overall, the number of diabetes‐related complications in metropolitan and non‐metropolitan cities increased from baseline over 2 years (Table S4C).

### 3.6 Anti‐diabetic treatment therapies

In 2 years, the total proportion of patients taking OAD + insulin increased (baseline: 24.0%, 1498/6234 vs. 2 years: 33.3%, 1917/5763), while the proportion of those taking only OADs, decreased (baseline: 74.4%, 4640/6234 vs. 2 years: 64.8%, 3735/5763) (Table S1).

Biguanides and sulfonylureas were the most prescribed OADs at baseline and 2 years (biguanides, baseline: 93.0%, 5798/6234 and in 2 years: 92.7%, 5340/5763; sulfonylureas, baseline: 76.3%, 4759/6234 and in 2 years: 77.7%, 4480/5763). The highest increase in OAD addition was seen for dipeptidyl peptidase 4 (DPP4) inhibitors (baseline: 48.9%, 3049/6234 vs. 2‐years: 61.2%, 3526/5763) followed by sodium‐glucose cotransporter‐2 inhibitors (baseline: 10.5%, 654/6234 vs. 2 years: 21.3%, 1227/5763) (Table S5). The mean (95% CI) change in HbA1c from baseline was −0.5 (−0.5, −0.4) in ≤3 OAD subgroup and −0.4 (−0.6, −0.2) in >3 OAD subgroup. Improvement in the glycaemic parameters in 2 years was more in the ≤3 OAD versus >3 OAD subgroup (*p* values were 0.8193, 0.1139 and 0.5541 for HbA1c, FPG and PPG, respectively) (Table 5).

**TABLE 5:** Comparison of glycaemic assessments between additional therapy groups for subgroup of patients who attended all visits (*N* = 6234).

	Change in HbA1c (%)^a^	Change in FPG (mg/dl)^a^	Change in PPG (mg/dl)^a^
	*n*; mean (95% CI)	*n*; mean (95% CI)	*n*; mean (95% CI)
Insulin‐naïve	1454; −0.4 (−0.5, −0.4)	1821; −9.9 (−12.1, −7.8)	1705; −17.2 (−20.6, −13.8)
Insulin	505; −1.0 (−1.2, −0.9)	721; −25.8 (−30.5, −21.0)	697; −38.7 (−45.2, −32.2)
*p*‐value^b^	<.0001	<.0001	<.0001
Basal (with/without prandial) insulin	234; −1.1 (−1.4, −0.9)	323; −26.0 (−32.9, −19.2)	323; −38.3 (−47.5, −29.0)
Premix (with/without prandial)	198; −1.0 (−1.3, −0.8)	298; −27.9 (−35.5, −20.4)	282; −37.3 (−47.9, −26.8)
*p*‐value^b^	.5758	.7125	.8915
Basal long‐acting (without prandial) insulin	174; −1.3 (−1.6, −1.0)	238; −23.8 (−31.5, −16.0)	230; −40.0 (−51.4, −28.7)
Premix (without prandial) insulin	205; −1.1 (−1.3, −0.8)	304; −27.7 (−35.3, −20.2)	289; −39.7 (−50.2, −29.3)
*p*‐value^b^	.2520	.4747	.9696
≤3 OAD	843; −0.5 (−0.5, −0.4)	1110; −10.9 (−13.5, −8.3)	1024; −17.4 (−21.6, −13.3)
>3 OAD	218; −0.4 (−0.6, −0.2)	262; −6.0 (−11.8, −0.1)	247; −14.6 (−23.1, −6.0)
*p*‐value^b^	.8193	.1139	.5541

Abbreviations: CI, confidence interval; FPG, fasting plasma glucose; HbA1c, glycated haemoglobin; OAD, oral anti‐diabetic; PPG, postprandial glucose.

^a^ Represents change from baseline to 2 years.

^b^ *p*‐value is calculated between the treatment subgroups using independent *t*‐test.

In 2 years, the commonly prescribed injectables were basal and premix insulins (basal insulin, baseline: 13.5%, 839/6234 and 2 years: 20.6%, 1188/5763; premix insulin, baseline: 11.0%, 683/6234 and 2 years: 14.7%, 849/5763) (Table S5). The change from baseline in all three glycaemic parameters in 2 years was significantly more in the insulin‐receiving subgroup than in the insulin‐naïve subgroup (*p* < .0001). The mean (95% CI) change in HbA1c from baseline was −1.0 (−1.2, −0.9) in insulin subgroup and −0.4 (−0.5, −0.4) in insulin‐naïve subgroup (*p* < .0001, both) (Table 5).

### 3.7 Adverse drug reactions

A total of 19 events (18 patients) of hypoglycaemia were recorded between the 1‐year to 18‐month period. In the latter 6 months of the 2‐year visit, 0.3% of patients (17/6234) reported hypoglycaemic events (documented symptomatic, *n* = 11; asymptomatic, *n* = 2; nocturnal, *n* = 4). (Table S3). One adverse drug reaction (hypoglycaemia) by one patient was noted until the end of 2 years.

## 4 DISCUSSION

This pan‐India, real‐world, large‐scale, longitudinal study is designed to assess glycaemic control, treatment trends, CV risks and development of macro‐ and microvascular complications over 3 years in Indian adults with T2DM. Herewith, we report an interim analysis done at the 2‐year time point. Over 2 years, while there was an overall improvement in glycaemic status, only 1 in 5 patients achieved HbA1c < 7%. Approximately one‐third of the patients in metropolitan (30.6%) and non‐metropolitan (35.4%) cities had HbA1c < 7% at the end of 2 years. While also highest at baseline among microvascular complications, the proportion of patients with neuropathy showed an increase at the end of 2 years. Hypertension and dyslipidaemia were the most reported CV risks. The 2‐year results show that the majority of the patients with T2DM are treated with OADs. Biguanides and sulfonylureas are the most commonly used OADs in Indian routine clinical practice.

In this study, most of the patients (~90%) were aged between 31 and 65 years and had obesity (67%). With age it is difficult to reduce weight as the deposition of central fat becomes more pronounced and obesity sets in. Obesity paves the way for lifestyle disorders, one of them being T2DM.^16^ Previous reports demonstrate that obesity is a well‐established risk factor for chronic illnesses like T2DM.^17^, ^18^Worsening of T2DM leads to CV risks and vascular complications.^6^, ^7^, ^8^, ^9^, ^10^ A possible mechanism linking T2DM and obesity with subsequent CV complications is inflammation and lipid accumulation due to overexpression of cytokines (tumour necrosis factor‐α, interleukin (IL)‐1, IL‐6, leptin, resist in monocyte chemoattractant protein (MCP)‐1, plasminogen activator inhibitor (PAI)‐1, fibrinogen and angiotensin) by adipose tissue, which have a deleterious effect on blood vessels and can lead to the development of MI and cardiomyopathy.^19^

Current treatment recommendations instate close monitoring and control of glycaemic levels to improve cardiac outcomes.^2^ However, a considerable gap exists between diabetes care followed in real practice versus that recommended by evidence‐based guidelines.^20^ The results of this study shed light on the real‐world burden of uncontrolled diabetes in India. In this 2‐year analysis, 20.8% of the study population had optimal glycaemic control (HbA1c < 7%). In the GOAL study, 29.7% of patients had glycaemic control after 12 months, and in the wave −7 of IDMPS study, 25.2% of patients had optimal glycaemic control.^21^, ^22^ The proportion of patients having optimum glycaemic control worsened over 2 years despite an increase in the use of OADs, reiterating the need for early control.

Despite improvement in the HbA1c levels in 0.6% of patients in 2 years, there was an overall increase in the number of patients with microvascular and macrovascular complications. Those patients who were overweight or had suboptimal glycaemic control or CV risk factors had more complications versus those without these comorbidities, thus, substantiating an established fact that high BMI and poor glycaemic control lead to vascular complications.^18^, ^19^, ^23^ The UKPDS 38 study examined the effect of tight control of blood pressure on macrovascular and microvascular complications in patients with T2DM. After 9 years of follow‐up, the results showed a 34% reduction in macrovascular complications (MI, sudden death, stroke and PVD) and a 37% reduction in the risk of microvascular complications (retinopathy requiring photocoagulation, vitreous haemorrhage and fatal or non‐fatal renal failure) in tightly controlled blood pressure group compared with the less tightly controlled group.^24^ In this study, neuropathy was the most reported complication in 2 years. These results are consistent with the observation of the 1‐year LANDMARC study^13^ and A1chieve study.^25^ It is a well‐known fact that in people with T2DM, uncontrolled high blood sugar for a long duration degenerates the neurons, leading to a loss of sensory function or diabetic neuropathy.^26^ Previous reports have established the observation that hypertension and dyslipidaemia are generally prevalent in people with diabetes.^27^, ^28^ The 2‐year data in the present study also showed that all three microvascular complications (neuropathy, nephropathy and retinopathy) and heart failure were reported in more patients with CV risks than without CV risks.

As anticipated with the progressive nature of the disease after 2 years in the study, half of the patients who had diabetes for >10 years at baseline were taking OAD + insulin and those who had diabetes for ≤10 years at baseline were predominantly on OADs. Biguanides and sulfonylureas continued to remain the most used OAD classes in 2 years. Similar to the 1‐year results, the highest addition was seen in patients on DPP4 inhibitors.^13^ There was a shift from the use of OADs towards the introduction of insulin as the need for injectables is common in people with longer duration of diabetes.^2^, ^30^ In 2 years, improvement in glycaemic parameters was significantly higher in the insulin receiving subgroup than in the insulin‐naïve subgroup (*p* < .0001), which is in alignment to the 1‐year results of the LANDMARC study.^13^ The ORIGIN study showed that the progression of diabetes was substantially reduced with timely insulin treatment in comparison with standard of care.^29^ There is also evidence that in patients who are diagnosed with severe hyperglycaemia (HbA1c > 9%–10%), insulin can control gluco‐ and lipo‐toxicity within a few days of therapy.^30^ Therefore, timely initiation and intensification of insulin treatment can help achieve glycaemic control and improve the treatment outcomes.

There was an improvement in the HbA1c levels in patients from both metropolitan and non‐metropolitan cities. The microvascular complications were significantly more in patients from non‐metropolitan cities than in metropolitan cities (*p* < .0001). The number of CV deaths and newly reported macrovascular complications were also higher in patients from non‐metropolitan cities than in metropolitan cities. Difference in the disease management between metropolitan and non‐metropolitan cities could result in variable outcomes. Further studies can help understand the diabetes management patterns in metropolitan and non‐metropolitan cities.

LANDMARC is one of the first‐of‐its‐kind large‐scale longitudinal studies from India involving 6234 patients from 382 centers to investigate microvascular and macrovascular complications, glycaemic control and therapy pattern in patients with T2DM over 3 years across India. This real‐world data analysis provides a longitudinal course of the T2DM burden, management practices and related complications across the nation including a sub‐analysis across metropolitan and non‐metropolitan cities of India. This study is inherent to the limitations associated with a real‐world study. Moreover, being observational in nature, any study‐specific procedures or screening for complications or CV risks were not possible. Furthermore, this study does not capture data on factors such as financial status, educational qualification of the patients and access to treatment facilities that warranted a better understanding, if investigated.

## 5 CONCLUSIONS

The results of the second year of the LANDMARC study showed high burden of uncontrolled diabetes in patients with T2DM from India. In 2 years, 17.6% of the study population had microvascular complications, predominantly neuropathy. A higher number of complications were observed in patients from non‐metropolitan versus metropolitan cities. Hypertension was the most reported CV risk. In 2 years, an increase in number of injectables was also observed. These 2‐year trends are similar to those observed in the 1‐year results of the LANDMARC study. This pan‐India, real‐world study highlights the need for effective diabetes management including enhanced awareness among patients and providers to meet glycaemic targets and prevent CV risk and vascular complications in a developing country like India with high prevalence of T2DM.

## AUTHOR CONTRIBUTIONS

**Ashok Kumar Das:** Conceptualization (equal); data curation (supporting); formal analysis (supporting); funding acquisition (supporting); investigation (lead); methodology (equal); project administration (supporting); resources (supporting); software (supporting); supervision (lead); validation (lead); visualization (lead); writing – original draft (equal); writing – review and editing (equal). **Sanjay Kalra:** Conceptualization (supporting); data curation (supporting); formal analysis (supporting); funding acquisition (supporting); investigation (equal); methodology (supporting); project administration (supporting); resources (supporting); software (supporting); supervision (equal); validation (equal); visualization (equal); writing – original draft (equal); writing – review and editing (equal). **Shashank R Joshi:** Conceptualization (supporting); data curation (supporting); formal analysis (supporting); funding acquisition (supporting); investigation (equal); methodology (supporting); project administration (supporting); resources (supporting); software (supporting); supervision (equal); validation (equal); visualization (equal); writing – original draft (equal); writing – review and editing (equal). **Ambrish Mithal:** Conceptualization (supporting); data curation (supporting); formal analysis (supporting); funding acquisition (supporting); investigation (equal); methodology (supporting); project administration (supporting); resources (supporting); software (supporting); supervision (equal); validation (equal); visualization (equal); writing – original draft (equal); writing – review and editing (equal). **Prasanna Kumar KM:** Conceptualization (supporting); data curation (supporting); formal analysis (supporting); funding acquisition (supporting); investigation (equal); methodology (supporting); project administration (supporting); resources (supporting); software (supporting); supervision (equal); validation (equal); visualization (equal); writing – original draft (equal); writing – review and editing (equal). **Ambika G Unnikrishnan:** Conceptualization (supporting); data curation (supporting); formal analysis (supporting); funding acquisition (supporting); investigation (equal); methodology (supporting); project administration (supporting); resources (supporting); software (supporting); supervision (equal); validation (equal); visualization (equal); writing – original draft (equal); writing – review and editing (equal). **Hemant Thacker:** Conceptualization (supporting); data curation (supporting); formal analysis (supporting); funding acquisition (supporting); investigation (equal); methodology (supporting); project administration (supporting); resources (supporting); software (supporting); supervision (equal); validation (equal); visualization (equal); writing – original draft (equal); writing – review and editing (equal). **Bipin Sethi:** Conceptualization (supporting); data curation (supporting); formal analysis (supporting); funding acquisition (supporting); investigation (equal); methodology (supporting); project administration (supporting); resources (supporting); software (supporting); supervision (equal); validation (equal); visualization (equal); writing – original draft (equal); writing – review and editing (equal). **Subhankar Chowdhury:** Conceptualization (supporting); data curation (supporting); formal analysis (supporting); funding acquisition (supporting); investigation (equal); methodology (supporting); project administration (supporting); resources (supporting); software (supporting); supervision (equal); validation (equal); visualization (equal); writing – original draft (equal); writing – review and editing (equal). **Amarnath Sugumaran:** Conceptualization (equal); data curation (equal); formal analysis (supporting); funding acquisition (equal); investigation (equal); methodology (equal); project administration (equal); resources (supporting); software (supporting); supervision (equal); validation (equal); visualization (equal); writing – original draft (equal); writing – review and editing (equal). **Senthilnathan Mohanasundaram:** Conceptualization (equal); data curation (equal); formal analysis (supporting); funding acquisition (equal); investigation (equal); methodology (equal); project administration (equal); resources (supporting); software (supporting); supervision (equal); validation (equal); visualization (equal); writing – original draft (equal); writing – review and editing (equal). **Shalini Kesav Menon:** Conceptualization (equal); data curation (equal); formal analysis (supporting); funding acquisition (equal); investigation (equal); methodology (equal); project administration (equal); resources (supporting); software (supporting); supervision (equal); validation (equal); visualization (equal); writing – original draft (equal); writing – review and editing (equal). **Vaibhav Salvi:** Conceptualization (supporting); data curation (equal); formal analysis (equal); funding acquisition (supporting); investigation (equal); methodology (equal); project administration (equal); resources (supporting); software (equal); supervision (equal); validation (equal); visualization (equal); writing – original draft (equal); writing – review and editing (equal). **Deepa Chodankar:** Conceptualization (equal); data curation (equal); formal analysis (lead); funding acquisition (supporting); investigation (equal); methodology (lead); project administration (equal); resources (lead); software (lead); supervision (equal); validation (equal); visualization (equal); writing – original draft (equal); writing – review and editing (equal). **Saket Thaker:** Conceptualization (supporting); data curation (supporting); formal analysis (supporting); funding acquisition (supporting); investigation (equal); methodology (supporting); project administration (supporting); resources (supporting); software (supporting); supervision (equal); validation (equal); visualization (equal); writing – original draft (equal); writing – review and editing (equal). **Chirag Trivedi:** Conceptualization (equal); data curation (equal); formal analysis (equal); funding acquisition (equal); investigation (equal); methodology (equal); project administration (equal); resources (equal); software (supporting); supervision (equal); validation (equal); visualization (equal); writing – original draft (equal); writing – review and editing (equal). **Subhash Kumar Wangnoo:** Conceptualization (supporting); data curation (supporting); formal analysis (supporting); funding acquisition (supporting); investigation (equal); methodology (supporting); project administration (supporting); resources (supporting); software (supporting); supervision (equal); validation (equal); visualization (equal); writing – original draft (equal); writing – review and editing (equal). **Abdul Zargar:** Conceptualization (supporting); data curation (supporting); formal analysis (supporting); funding acquisition (supporting); investigation (equal); methodology (supporting); project administration (supporting); resources (supporting); software (supporting); supervision (equal); validation (equal); visualization (equal); writing – original draft (equal); writing – review and editing (equal). **Nadeem Rais:** Conceptualization (supporting); data curation (supporting); formal analysis (supporting); funding acquisition (supporting); investigation (equal); methodology (supporting); project administration (supporting); resources (supporting); software (supporting); supervision (equal); validation (equal); visualization (equal); writing – original draft (equal); writing – review and editing (equal).

## FUNDING INFORMATION

This study was funded by Sanofi.

## CONFLICT OF INTEREST

AKD, AM, AGU and NR received honoraria from Sanofi and other pharmaceutical companies. KMPK is on the advisory board of Sanofi and received an honorarium for his talks. SJ received speaker/advisory/research grants from Abbott, AstraZeneca, Biocon, Boehringer Ingelheim (BI), Eli Lilly, Franco Indian, Glenmark, Lupin, Marico, MSD, Novartis, Novo Nordisk, Roche, Sanofi, Serdia, Twinhealth and Zydus. SK received honoraria/speaker fees from Eli Lilly, Novo Nordisk and Sanofi. HT received honoraria from MSD, Novartis, Sanofi and other companies for advice and lectures. BS received an honorarium from Aventis, Novo Nordisk, Eli Lilly, BI and MSD. SC received honoraria/grants from Biocon, BI, Intas, Novartis, Sanofi and Serdia. SKW has nothing to declare. AHZ received honoraria from Novo Nordisk, Eli Lilly, Johnson & Johnson, AstraZeneca, BI and Sanofi. AS, SM, SKM, DC, VS, ST and CT are employees of Sanofi and may hold stock options.

## PREVIOUS PRESENTATIONS AND PUBLICATIONS

Part of the data from this paper was presented at the 82nd Scientific Sessions of American Diabetes Association (ADA) 2022, New Orleans, LA, and the 58th Annual Meeting of the European association for the study of Diabetes, Stockholm, 19–23 September 2022. The protocol of this study is published in *Diabetic Medicine*; DOI: 10.1111/dme.14171. The baseline data and 1‐year data of this study have been published in *Endocrinology, Diabetes & Metabolism*; their DOIs are 10.1002/edm2.231 and 10.1002/edm2.316, respectively.

## Supporting information


Table S1.
Table S2.
Table S3.
Table S4.
Table S5.

## Data Availability

Qualified researchers may request access to person‐level data and related study documents including the clinical study report, study protocol with any amendments, blank case report form, statistical analysis plan and data set specifications. Person‐level data will be anonymized, and study documents will be redacted to protect the privacy of study patients. Further details on Sanofi's data sharing criteria, eligible studies and the process for requesting access can be found at https://vivli.org/.
