# Supplementary material for: Two‐year trends from the LANDMARC study: A 3‐year, pan‐India, prospective, longitudinal study on the management and real‐world outcome in patients with type 2 diabetes mellitus
Source: Endocrinol Diabetes Metab. 2023 Feb 1;6(2):e404. doi: 10.1002/edm2.404 (PMC10000633; doi:10.1002/edm2.404)
Supplement: Supplementary file 1 — Table S1. Table S2. Table S3. Table S4. Table S5. [file EDM2-6-e404-s001.docx]

# Supporting information

# Supplementary tables

**Table S1: Diabetes treatment stratified by diabetes duration at baseline and 2-year**

| **Duration of diabetes** | **2–5 years** | | **6-10 years** | | **>10 years** | | **Total** | |
| --- | --- | --- | --- | --- | --- | --- | --- | --- |
|  | **Baseline**  **N=2359**  **n (%)** | **2-year**  **N=2164**  **n (%)** | **Baseline**  **N=2147**  **n (%)** | **2-year**  **N=2012**  **n (%)** | **Baseline**  **N=1728**  **n (%)** | **2-year**  **N=1587**  **n (%)** | **Baseline**  **N=6234**  **n (%)** | **2-year**  **N=5763**  **n (%)** |
| Only on OAD | 2007 (85.1) | 1654 (76.4) | 1641 (76.4) | 1335 (66.4) | 992 (57.4) | 746 (47.0) | 4640 (74.4) | 3735 (64.8) |
| Only on Insulin | 8 (0.3) | 10 (0.5) | 5 (0.2) | 7 (0.3) | 13 (0.8) | 18 (1.1) | 26 (0.4) | 35 (0.6) |
| OAD + Insulin | 331 (14.0) | 479 (22.1) | 477 (22.2) | 636 (31.6) | 690 (39.9) | 802 (50.5) | 1498 (24.0) | 1917 (33.3) |
| OAD + non-insulin injectables | 11 (0.5) | 13 (0.6) | 16 (0.7) | 16 (0.8) | 18 (1.0) | 8 (0.5) | 45 (0.7) | 37 (0.6) |
| Insulin + non-insulin injectables | 0 | 1 (0.0) | 0 | 1 (0.0) | 0 | 0 | 0 | 2 (0.0) |
| OAD + Insulin + non-insulin injectables | 2 (0.1) | 7 (0.3) | 8 (0.4) | 17 (0.8) | 15 (0.9) | 13 (0.8) | 25 (0.4) | 37 (0.6) |
| Values are presented as n (%) unless specified otherwise.  Note: Percentages are based on the number of patients using at least one anti-diabetic drug at the corresponding visit within each diabetes duration group. Total number of patients (N=5763) are the ongoing patients in 2 years.  N, number of patients analyzed; n, number of patients with non-missing results at the visit; OAD, oral anti-diabetic drugs. | | | | | | | | |

**Table S2: Microvascular and macrovascular complications in 2 years, by BMI, HbA1c and CV risk factors (N=6234)**

| **Complications** | **BMI <23 kg/m^2^ n (%)** | **BMI ≥23 kg/m^2^ n (%)** | **p-value*** | **HbA1c <7 % n (%)** | **HbA1c ≥7 % n (%)** | **p-value*** | **With CV risk factors n (%)** | **Without CV risk factors n (%)** | **p-value*** |
| --- | --- | --- | --- | --- | --- | --- | --- | --- | --- |
| Neuropathy | 103 (1.7) | 586 (9.4) | 0.7238 | 166 (2.7) | 383 (6.1) | 0.0932 | 665 (10.7) | 233 (3.7) | <0.0001 |
| Nephropathy | 29 (0.5) | 128 (2.1) | 0.2782 | 38 (0.6) | 107 (1.7) | 0.0624 | 185 (3.0) | 36 (0.6) | <0.0001 |
| Retinopathy | 22 (0.4) | 85 (1.4) | 0.1346 | 21 (0.3) | 70 (1.1) | 0.0351 | 130 (2.1) | 35 (0.6) | <0.0001 |
| MI | 0 | 1 (0.0) | >0.9999 | 1 (0.0) | 0 | 0.3337 | 3 (0.1) | 7 (0.1) | 0.1249 |
| Stroke | 0 | 1 (0.0) | >0.9999 | 0 | 1 (0.0) | >0.9999 | 2 (0.0) | 0 | 0.5049 |
| PVD | 0 | 1 (0.0) | >0.9999 | 1 (0.0) | 3 (0.1) | >0.9999 | 4 (0.1) | 0 | 0.1324 |
| ACS | 0 | 1 (0.0) | >0.9999 | 1 (0.0) | 0 | 0.3337 | 1 (0.0) | 0 | >0.9999 |
| Heart failure | 3 (0.1) | 14 (0.2) | 0.7372 | 6 (0.1) | 8 (0.1) | 0.4506 | 20 (0.3) | 3 (0.1) | 0.0024 |
| Unstable angina | 0 | 1 (0.0) | >0.9999 | 1 (0.0) | 0 | 0.3337 | 1 (0.0) | 0 | >0.9999 |
| Values are presented as n (%) unless specified otherwise.  *****p-values are reported from Fisher’s test if the cell frequency is lesser than 5. p-values are reported using the Chi Square test otherwise. The null hypothesis is that there is no difference between the two populations’ proportions. The p-values reported are not adjusted for inflation in Type I error.  ACS, acute coronary syndrome; BMI, body mass index; CV, cardiovascular; HbA1c, glycated hemoglobin; MI, myocardial infarction; N, number of patients analyzed; n, number of patients with non-missing results at the visit; PVD. peripheral vascular disease | | | | | | | | | |

**Table S3: Hypoglycemic events, revascularization procedure and hospitalization**

|  | **Total N=6234** | | | |
| --- | --- | --- | --- | --- |
|  | **1-year to 18-months** | | **18-months to 2-year** | |
|  | **n (%)** | **Ne** | **n (%)** | **Ne** |
| **Hypoglycemic events** | 18 (0.3) | 19 | 17 (0.3) | 17 |
| Severe hypoglycemia | 1 (0.0) | 1 | 0 | 0 |
| Documented symptomatic hypoglycemia | 9(0.1) | 9 | 11 (0.2) | 11 |
| Asymptomatic hypoglycemia | 4 (0.1) | 4 | 2 (0.0) | 2 |
| Nocturnal hypoglycemia | 5 (0.1) | 5 | 4 (0.1) | 4 |
| **Revascularization Procedures** | 3 (0.0) | 3 | 1 (0.0) | 1 |
| **Hospitalization** | 3 (0.0) | 3 | 1 (0.0) | 3 |
| Non-fatal myocardial infarction | 1 (0.0) | 1 | 0 | 0 |
| Acute coronary syndrome | 2 (0.0) | 2 | 1 (0.0) | 1 |
| Heart failure | 0 | 0 | 1 (0.0) | 1 |
| Unstable angina | 0 | 0 | 1 (0.0) | 1 |
| Values are presented as n (%) unless specified otherwise.  Percentages are based on the number of patients within each subgroup in the eligible population. Eligible population includes all patients who have met inclusion/exclusion criteria for the study.  N, total number of patients analyzed; n, number of patients with hypoglycemic events/revascularization procedures/hospitalization; Ne, number of hypoglycemic/ revascularization procedures/hospitalization events | | | | |

**Table S4A: Patient characteristics and glycemic trends in 2 years in metropolitan versus non-metropolitan cities**

| **Parameters** | | **Metropolitan cities**  **(N=2376)** | **Non-Metropolitan cities**  **(N=** **3858)** |
| --- | --- | --- | --- |
| **Age, at baseline** | Year, mean ± SD | 52.4 ± 9.3 | 52.0 ± 9.0 |
| **Duration of type 2 diabetes mellitus, at baseline** | Year, mean ± SD | 8.6 ± 5.7 | 8.6 ± 5.6 |
| **HbA1c (%), at baseline** | n, mean ± SD | 1804, 8.1 ± 1.6 | 2673, 8.0 ± 1.6 |
| **HbA1c (%)** | 2-Year, n | 1607 | 2280 |
|  | mean ± SD | 7.5 ± 1.1 | 7.4 ± 1.1 |
|  | Change from baseline,  n, mean (95% CI) | 1013, -0.5 (-0.6, -0.4) | 1209, -0.7 (-0.8, -0.6) |
|  | P value* | 0.0450 | |
| **FPG (mg/dL)** | 2-Year, n | 1727 | 2593 |
|  | mean ± SD | 127.1 ± 34.0 | 126.1 ± 32.5 |
|  | Change from baseline,  n, mean (95% CI) | 1244, -17.2 (-20.2, -14.1) | 1674, -15.2 (-17.8, -12.6) |
|  | P value* | 0.3408 | |
| **PPG (mg/dL)** | 2-Year, n | 1658 | 2452 |
|  | mean ± SD | 182.0 ± 51.8 | 181.5 ± 49.9 |
|  | Change from baseline,  n, mean (95% CI) | 1143, -24.4 (-29.0, -19.9) | 1615, -25.7 (-29.5, -21.8) |
|  | P value* | 0.6890 | |

**Table S4B: Microvascular and Macrovascular complications in 2 years in metropolitan versus non-** **metropolitan cities**

| **Parameters** | **Metropolitan cities**  **(N=2376)**  **n (%)** | **Non-metropolitan cities**  **(N=3858)**  **n (%)** |
| --- | --- | --- |
| **Microvascular complications** | **290 (4.7)** | **806 (12.9)**** |
| Neuropathy | 235 (3.8) | 663 (10.6)****** |
| Nephropathy | 47 (0.8) | 174 (2.8)****** |
| Retinopathy | 34 (0.6) | 131 (2.1)****** |
| **Macrovascular complications** | **6 (0.1)** | **33 (0.5)***** |
| MI† | 3 (0.1) | 7 (0.1) |
| Stroke† | 0 | 2 (0.0) |
| PVD† | 0 | 4 (0.1) |

**Table S4C: Microvascular and macrovascular complications by metropolitan versus non-** **metropolitan cities**

| **Parameters** | **Metropolitan cities**  **(N=2376)**  **n (%)** | | **Non-metropolitan cities**  **(N=3858)**  **n (%)** | | |
| --- | --- | --- | --- | --- | --- |
| **Microvascular complications at 2-year, Ne** | | **316** | | **968** |  |
| **Patients with** **microvascular complications** | | **290 (12.2)** | | **806 (20.9)** |  |
| Neuropathy | | 235 (81.0) | | 663 (82.3) |  |
| Nephropathy | | 47 (16.2) | | 174 (21.6) |  |
| Retinopathy | | 34 (11.7) | | 131 (16.3) |  |
| **New microvascular complications at 2-year, Ne** | | **16** | | **41** |  |
| **Patients with new microvascular complications, n** | | **15 (0.6)** | | **40 (1.0)** |  |
| Neuropathy | | 10 (0.5) | | 22 (0.7) |  |
| Nephropathy | | 3 (0.1) | | 16 (0.4) |  |
| Retinopathy | | 3 (0.1) | | 3 (0.1) |  |
| **Macrovascular complications at 2-year, Ne** | | **70** | | **135** |  |
| **Patients with macrovascular complications, n** | | **68 (2.9)** | | **127 (3.3)** |  |
| MI | | 30 (44.1) | | 50 (39.4) |  |
| Stroke | | 12 (17.6) | | 21 (16.5) |  |
| CV death | | 5 (7.4) | | 25 (19.7) |  |
| PVD | | 23 (33.8) | | 38 (29.9) |  |
| No complication | | - | | - |  |
| Unknown | | - | | - |  |
| **New macrovascular complications at 2-year, Ne** | | **1** | | **14** |  |
| **Patients with new macrovascular complications, n** | | **1 (0.0)** | | **14 (0.4)** |  |
| MI | | 0 | | 1 (0.0) |  |
| Stroke | | 0 | | 1 (0.0) |  |
| CV death | | 1 (0.0) | | 8 (0.2) |  |
| PVD | | 0 | | 4 (0.1) |  |

Metropolitan cities include Bengaluru, Chennai, Delhi, Hyderabad, Kolkata, and Mumbai.

Duration of T2DM (years) = Informed consent date − Start date of T2DM.

*****p-values are reported using an independent t-test with the null hypothesis that the mean change from baseline in glycemic status is equal in the two groups. The p-values reported are not adjusted for inflation in Type I error.

**p-values are reported from Fishers test if the cell frequency is lesser than 5. p-values are reported using the Chi Square test otherwise. The null hypothesis is that there is no difference between the two population proportions. The p-values reported are not adjusted for inflation in Type I error. **p<0.0001; ***p=0.0034

†Complications are part of the definition for the primary endpoint.

Note: This is an interim analysis and possible modifications on variables and data could be performed for the subsequent interim analyses and the final analysis

CI, confidence interval; CV, cardiovascular; FPG, fasting plasma glucose; HbA1c, glycated hemoglobin; N, number of patients analyzed; Ne, number of events; n, number of patients with non-missing results; MI, myocardial infarction; PPG, postprandial glucose; PVD, peripheral vascular disease; SD, standard deviation; T2DM, type 2 diabetes mellitus.

**Table S5: Comparison of the use of oral and injectable glucose-lowering drugs at baseline and 2-year**

| **Anti-diabetic drug category** | **Baseline**  **N=6234**  **n (%)** | **2-year**  **N=5763**  **n (%)** |
| --- | --- | --- |
| **Oral anti-diabetic drugs** | 6208 (99.6) | 5726 (99.4) |
| Biguanides | 5798 (93.0) | 5340 (92.7) |
| Sulfonylureas | 4759 (76.3) | 4480 (77.7) |
| DPP-IV inhibitors | 3049 (48.9) | 3526 (61.2) |
| Thiazolidinediones | 698 (11.2) | 814 (14.1) |
| Alpha-glucosidase inhibitors | 1161 (18.6) | 1453 (25.2) |
| Meglitinides | 59 (0.9) | 64 (1.1) |
| Sodium glucose cotransporter 2 inhibitors | 654 (10.5) | 1227 (21.3) |
| Anti-malarial drug | 2 (0.0) | 7 (0.1) |
| **Injectable glucose lowering drugs** | 1594 (25.6) | 2028 (35.2) |
| GLP-1 analogs | 70 (1.1) | 76 (1.3) |
| Basal Insulin | 839 (13.5) | 1188 (20.6) |
| Prandial Insulin | 231 (3.7) | 327 (5.7) |
| Premix Insulin | 683 (11.0) | 849 (14.7) |
| Values are presented as n (%) unless specified otherwise.  DPP-IV, dipeptidyl peptidase-IV; GLP-1, glucagon-like peptide-1; N, number of patients analyzed; n, number of patients with non-missing results at the visit | | |
